# An anatomical feasibility study using CTA reconstruction for modified percutaneous lumbar vertebroplasty

**DOI:** 10.1186/s12891-022-05586-1

**Published:** 2022-07-21

**Authors:** Jianbiao Xu, Shali Fan, Yu Ni, James Reeves Mbori Ngwayi, Daniel Edward Porter, Jun Guo

**Affiliations:** 1 School of Clinical Medicine, Tsinghua University, Beijing, China; 2 Orthopaedics Department, First Affiliated Hospital of Tsinghua University, Beijing, China; 3 Radiology Department, First Affiliated Hospital of Tsinghua University, Beijing, China

**Keywords:** Osteoporotic vertebral compression fracture, Percutaneous vertebroplasty, Lumbar arteries

## Abstract

**Background:**

Lumbar vertebroplasty via several different types of extrapedicular approach has been reported with acceptable clinical results yet the anatomical basis for its safety is not fully explored. Injury to the lumbar arteries (LAs) is one of the most important potential complications. However, anatomical research on the course and variability of this structure is lacking. To investigate the anatomical feasibility of percutaneous vertebroplasty for lumbar osteoporotic vertebral compression fractures via a unilateral Extrapedicular approach.

**Methods:**

A total of 300 LAs of 30 patients with non-spinal disorders were retrospectively analyzed by computed tomographic angiography (CTA). The lateral aspect of the vertebral body was divided into 9 zones of approximately equal area. The anatomy and orientation of LAs were analyzed in detail.

**Results:**

LAs were most commonly found in the middle third of the body (zones 4, 5, and 6); the upper 1/3 of the vertebral body had LAs distributed only anteriorly and laterally (zones 1 and 2). No arteries were observed in the postero-superior segment (zone 3). From L1 to L3 an arched pattern predominated. At L4 an inferior oblique pattern (antero-superior to postero-inferior) predominated. Limited CTA visualization at L4 and particularly L5 as well as greater anatomical variation means that there is more uncertainty at these levels.

**Conclusion:**

From L1 to L3, the posterior superior segment (zone 1) of the vertebral body appears to be a safe area with low risk of arterial injury. This has relevance for design of a safe lumbar vertebral extrapedicular approach.

## Introduction

Percutaneous vertebroplasty (PVP) is a minimally invasive technique for the treatment of osteoporotic vertebral compression fractures (OVCF) [1, 2]. The procedure can quickly relieve pain, promote early mobilisation and reduce related complications caused by bed rest. Currently, the transpedicular approach is the most widely used surgical method for lumbar OVCF [3]. However, due to the restricted pedicle orientation, unilateral puncture has the disadvantage of uneven and usually ipsilateral distribution of bone cement, especially where the pedicle is narrow and following anatomical distortion due to fracture [4]. Uneven distribution of bone cement will affect the mechanical balance of the spine and the clinical efficacy of surgery [5].

Previous studies have shown that extrapedicular PVP puncture has the advantages of more uniform distribution of bone cement and reduced fluoroscopic exposure [6–10]. However, reports of damage to the lumbar artery during extrapedicular PVP puncture exist [11, 12]. Data on standard and anomalous arterial course relevant to this technique are not available, in part due to variation in the mode of extrapedicular approach described by different authors. The point of bone contact and cortical entry point on the lateral wall of the pedicle or vertebral body has not been described in detail. Integral to the safety of this approach is an understanding of the anatomical relationship between the lateral wall of the vertebral body and the lumbar arteries (LAs).

The purpose of this study, therefore, was to analyze the course of the lumbar segmental artery adjacent to the lateral wall of the vertebral body together with its variation, and to consider its clinical significance.

## Materials and methods

### Patients and general information

The imaging data of 30 adult patients with intra-abdominal or urological disorders undergoing total abdominal CTA (computerized tomography angiography) examination in our medical institution performed between 1 October 2019 and 1 December 2020. Inclusion criteria were as follows: (1) age was between 20–80 years old; (2) CTA reconstruction segment was T12-L5. Exclusion criteria: (1) patients with scoliosis, spinal tumor, and other serious lumbar diseases; (2) patients with previous lumbar surgery; (3) patients who met the inclusion criteria but had poor reconstruction image quality. This project was approved by the research ethics committee of our institution (Ethics approval NO: 2020–33-R01).

**Fig. 1:**
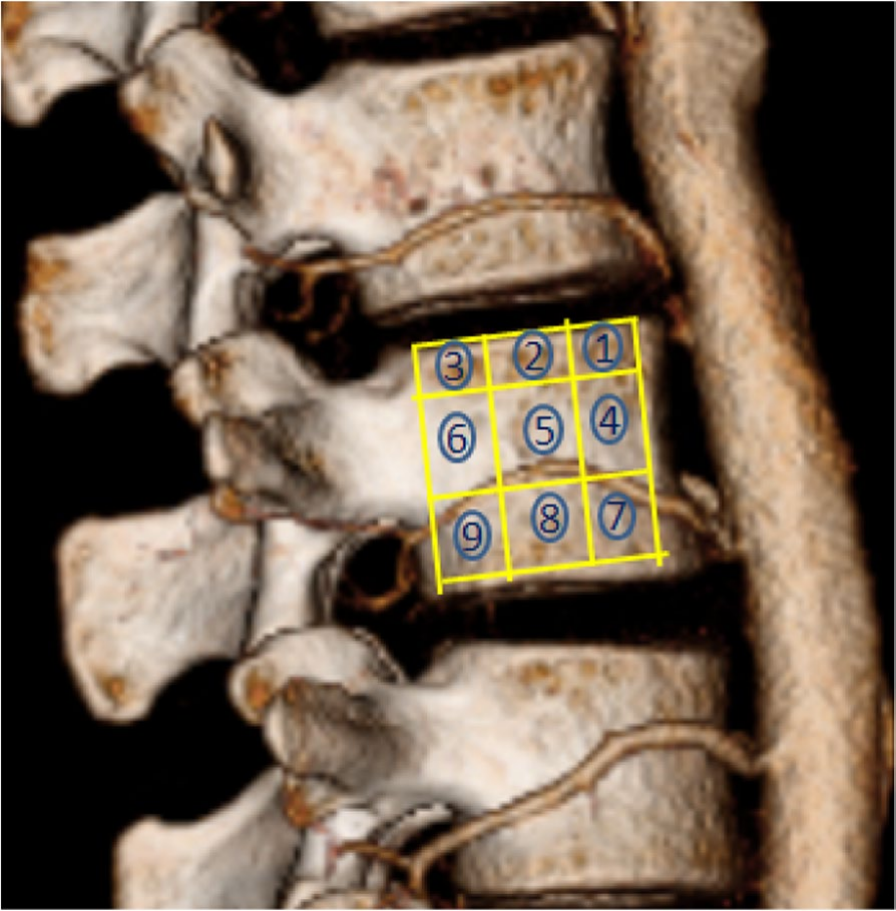
Schematic diagram of zones 1–9 on the on the lateral side of vertebral body

**Fig. 2:**
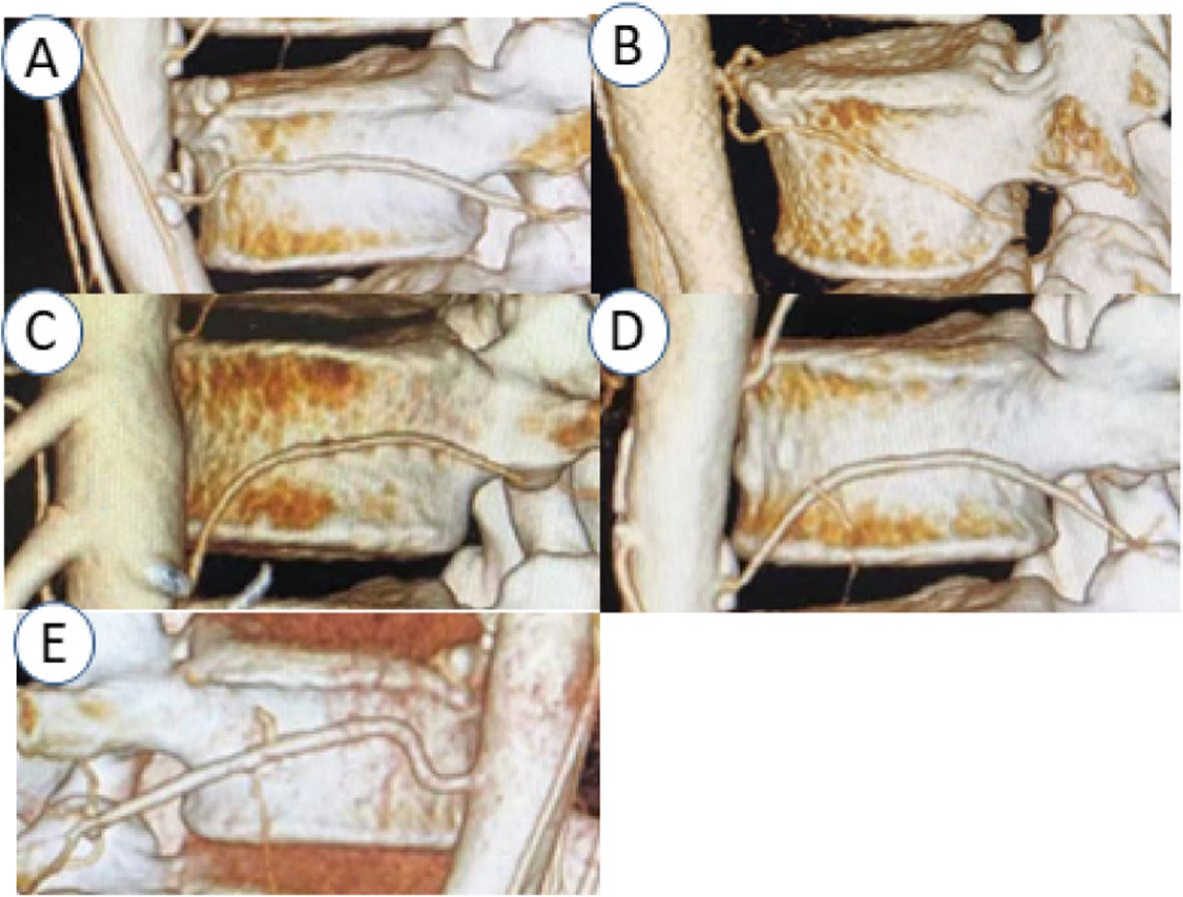
Classification of lumbar artery (LA) types. According to their course, LAs were divided into five types: **A** horizontal type; **B** inferior oblique type; **C** superior oblique type; **D** arch type; **E** mixed type

A Philips Brillance 64 spiral CTscanner (Amsterdam, Netherlands) was used to image the abdomen as described above.Images included the entire lower thoracic and lumbar spine, and associated arteries including LAs. Contrast agent (lomeron, 350 ml/ml) was injected into the median cubital vein at a rate of 5 ml/s and a dose of 80 ml. The scanning time was triggered at radiodensity threshold 150 Hounsfield Units. The thickness of CTA was 5 mm, the thickness of reconstruction was 1 mm, and the interval was 1 mm. The anatomical structure of each LA was reconstructed and evaluated on the Philips extended brilliance workspace (FUJIFILM Synapse 3D, Tokyo, Japan). First, a single researcher (XJB) extracted both left lateral and right lateral 2D images of the region of interest. Upon the image a grid was superimposed which divided, the vertebral body into nine zones;in the horizontal plane the middle (para-pedicular) third was defined as the zone between horizontal lines parallel to the upper and lower margins of the pedicle. the upper and lower thirds (supra-pedicular and infra-pedicular respectively) were defined as the portion of the body above and below this section. The image was divided further by two verical lines which produced three vertical segments of equal width from anterior to posterior. Second,the course of the LA across the surface of different segments was evaluated (Fig. 1). The evaluation was conducted by two doctors (XJB and FSL) independently, with the assistance of researcher GJ in case of disagreement. Finally, the segmental level, number, orientation, and branches of each LA was documented, and if not seen, its absence. According to their course, LAs were divided into five types: A. horizontal type; B. inferior oblique type (course from superanterior to posteroinferior); C. superior oblique type (course from inferoanterior to superoposterior); D. arched type; E. mixed type (Fig. 2).

## Statistical analysis

SPSS 23.0 (IBM, USA) was used for statistical analysis. Overall summary statistics were calculated in terms of frequencies and percentages for categorical variables and means ± SD for continuous variables. In this study, the t test was used for continuous data and chi square test for categorical data, with *P* < 0.05 as the significance standard.

## Results

### Demographics

Imaging data from 30 patients were collated; 18 males (age range 22–69 years, mean age 55.3 ± 25.7 years); 12 females (age range 48–72 years, mean age 57.2 ± 21.4 years). There was no significant difference in age between men and women (*P* > 0.05).

### Lumbar artery distribution

**Fig. 3:**
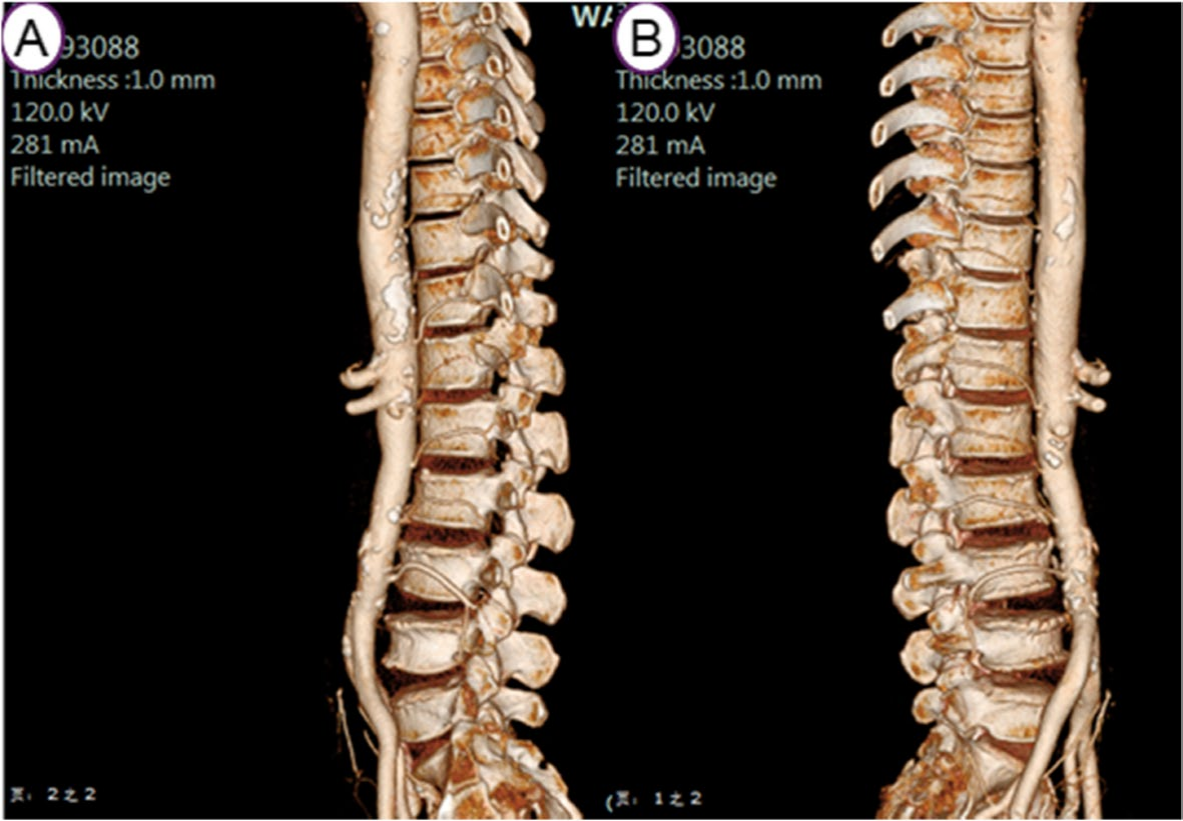
Distribution of LAs on both sides of vertebral body, **A** show Right LAs; **B** show Left LAs

**Fig. 4:**
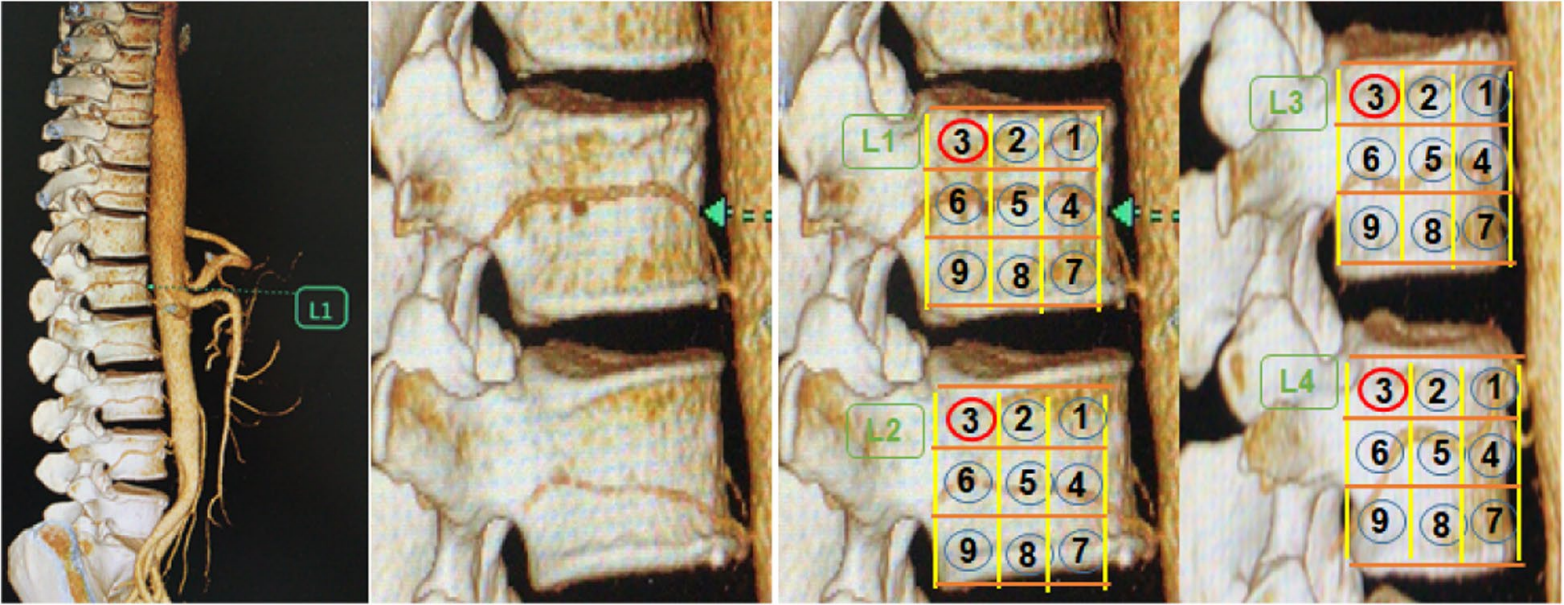
Distribution of LA in different lumbar segments (L1-L4) (L1: 4,5,6; L2: 4,5,6; L3: 4,5,6,7; L4: 1,4,5,6,9; L5 deletion)

Distribution of arteries within zones are recorded in Table 1. Typical examples of CTA and distribution zones of lumbar artery are shown in Figs. 3 and 4. For the upper three lumbar segments (L1-L3), LAs were found to cross mainly the para-pedicular and infrapedicular thirds of the vertebral body. No vessels were found to cross zone 3 (postero-superior aspect). The distribution of LAs in L4 and L5 were different, with the LA tending to run from antero-superior to postero-inferior (inferior oblique pattern – see below) (Table 1).

**Table 1:** Distribution of lumbar arteries within zones at each lumbar level L1-L5

Lumbar level	Supra-pedicular 1/3			Para-pedicular 1/3			Infrapedicular 1/3		
	Zone 1 (Anterior)	Zone 2(Middle)	Zone 3 (Posterior)	Zone 4 (Anterior)	Zone 5 (Middle)	Zone 6 (Posterior)	Zone 7 (Anterior)	Zone 8 (Middle)	Zone 9 (Posterior)
L1	0	0	0	25	51	50	48	6	12
L2	0	0	0	15	43	45	50	16	19
L3	8	2	0	35	54	35	33	5	28
L4	36	10	0	17	41	22	3	1	36
L5	1	1	0	1	1	0	3	2	3

**Fig. 5:**
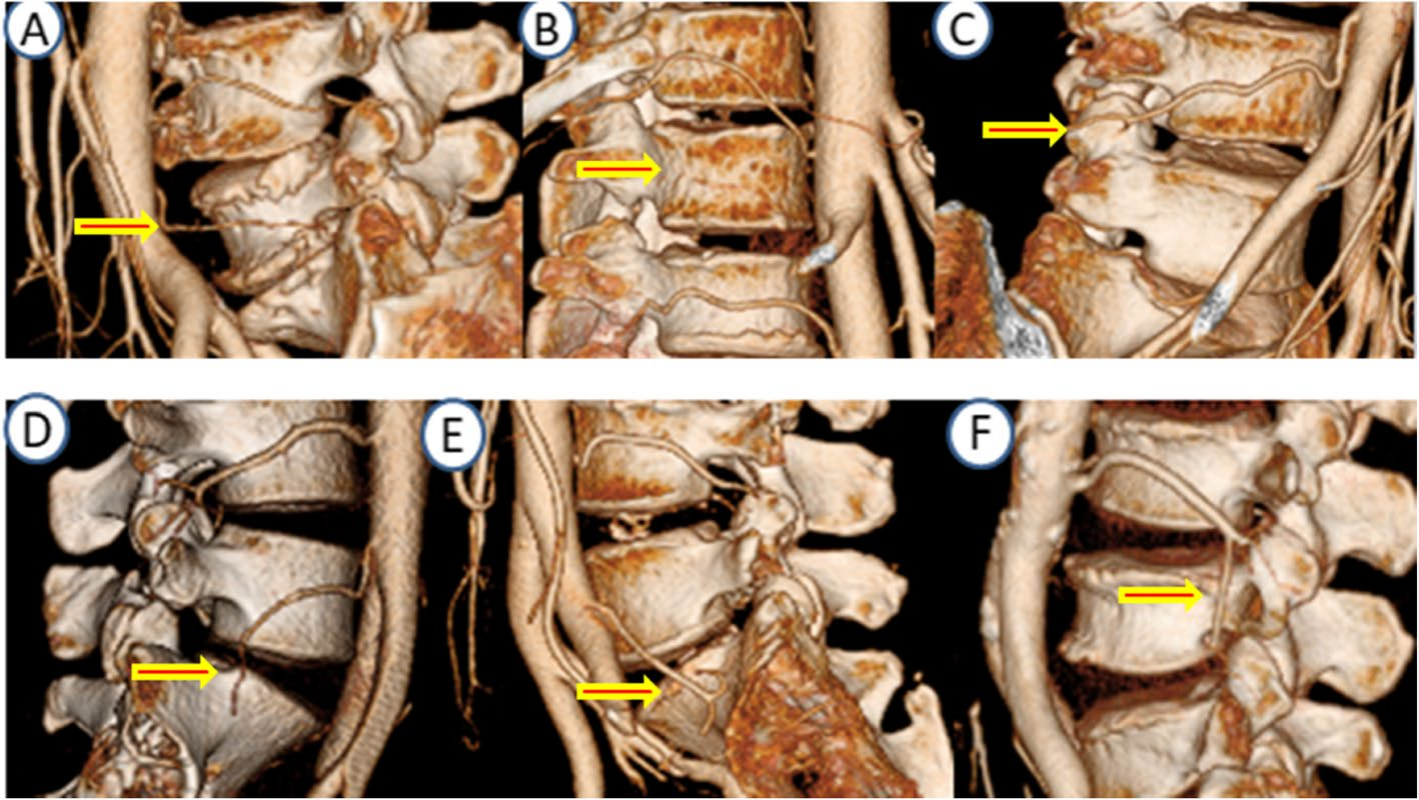
Rare distribution variations of lumbar artery Fig. **A **left L5 artery (Zone 1,5,9); **B **Absence of L1 artery; **C **L4 artery straddles L5 superior articular process obliquely; **D **Right L5 artery exists and straddles S1 vertebral body obliquely (Zone 1,5,9); **E **Left L5 artery exists and straddles S1 vertebral body obliquely (Zone 4,7); **F **L3 artery straddles L4 vertebral body obliquely

Twenty four spines had bilateral missing lumbar arteries on CTA. These were all at the L5 level where the absence rate was 80%; Ten spines had a unilateral missing lumbar artery (two at L1, two at L4 and 6 at L5. Where absent at one level, eight lumbar arteries from L3 to L5 demonstrated segmental branches above or below the missing vessel. Vessel absence or segmental braching were evenly distributed on left and right sides. No vessel was observed in zone 3 (postero-superior zone). The distribution of rare LA variations is shown in Fig. 5.

### Vascular classification

Among the 300 lumbar segments, we found 237 identifiable vascular patterns; 105 vessels were type D, 47 vessels were type A, 45 vessels were type B, 26 vessesl were type C and 14 vessels were type E. From L1 to L3 type D (arch) predominated), however at L4 type B (inferior oblique) predominated (Table 2).

**Table 2:** Distribution of lumbar artery vascualr patterns

N	Type A (horizontal)	Type B (inferior oblique)	Type C (superior oblique)	Type D (arch)	Type E (mixed)
L1					
Left	5	0	3	18	2
Right	7	0	11	12	0
Total	12	0	14	30	2
L2					
Left	9	0	1	19	0
Right	10	1	9	10	0
Total	19	1	10	29	0
L3					
Left	3	5	0	20	2
Right	8	2	2	15	3
Total	11	7	2	35	5
L4					
Left	1	18	0	6	2
Right	2	15	0	5	5
Total	3	33	0	11	7
L5					
Left	1	3	0	0	0
Right	1	1	0	0	0
Total	2	4	0	0	0
Total	47	45	26	105	14

## Discussion

To date, several series of patients with thoraco-lumbar extrapedicular approach have been reported without major complication [7–9, 12, 13]. Injury to the lumbar artery is one of the main concerns in extrapedicular puncture and has been reported in the literature [11]. However, this should be considered in the context of similar reports in transpedicular vertebroplasty. Five cases of lumbar artery injury caused by PVP orPKP have been reported in the previous literature. Among these, four cases were punctured through the transpedicular approach and 1 case was punctured through an extrapedicular approach (Table 3).Previous studies on the anatomy of the lumbar artery have focused on the morphology of the arteries in relation to the safety of anterior approaches [14] or the soft-tissue distribution of arteries around the spinal nerve with the aim of avoiding neurological symptoms during endoscopic surgery [15]. It is generally considered that surgery adjacent to the lumbar intervertebral disc will avoid injury to the lumbar artery in oblique lumbar interbody fusion (OLIF) or extreme lateral interbody fusion (XLIF) [16, 17]. To our knowledge, there are no studies on the safe area for extrapedicular PVP puncture, especially the distribution of blood vessels around the pedicle. This study is the first report to study the distribution of the lumbar artery on the lateral side of the vertebral body in relation to safe zones for percutaneous approaches.

**Table 3:** Reported cases of Lumbar artery injury caused by PVP or PKP

Case	Author	Disease	Operation	Damage location	Early symptoms	Diagnosis	Treatment	Outcome
Case1	Biafora,S.J.,et al.[18]	OVCF(L5)	PKP (Transpedicular approach)	Right L4LA	Incisional bleeding	Angiography	TAE	Cured
Case2	Heo, D.H.et al.[11]	OVCF(L2)	PVP (Extrapedicular approach)	Left L2 LA	Backache, left lower limb neural symptom, acute hypotension	Enhanced MRI	TAE	Cured
Case3	Puri et al.[19]	OVCF(L3)	PVP (Transpedicular approach)	Left L3LA	Backache, psoas hematoma	CTA	TAE	Cured
Case4	Puri et al.[19]	OVCF(L3)	PVP (Transpedicular approach)	Right L3LA	Anemia	CTA	TAE	Cured
Case5	Giordano et al.[20]	Vertebral Metastases	PVP (Transpedicular approach)	Left L2LA	Left lower abdominal pain	Contrast-enhanced CT	TAE	Cured

*PKP* Percutaneous Kyphoplasty, *PVP* Percutaneous Vertebroplasty, *CTA* Computed Tomography Angiography, *TAE* Transarterial Embolization

A thorough understanding of the spinal arteries before percutaneous endoscopic lumbar discectomy is a necessary stage to reduce the risk of intraoperative bleeding. In the present study, the distribution of the lumbar artery has the following characteristics: 1. the distribution of LAs in different vertebral segments is slightly different. L1, L2, and L3 were mainly distributed in the middle (para-pedicular) and lower (infra-pedicular) thirds; L4 was mainly distributed in the middle and upper (supra-pedicular) thirds, with a predominant inferior oblique pattern. Orita et. al. retrospectively analyzed LAs using MRI and shown that the LA branch angles are acute (< 90^。^) at L1-L3 and blunt (> 90) at L4-L5 [21]. However, the clarity of MRI displaying LAs is limited. Tezuka, F., et. al. analyzed 323 CTA images and found that from L1 to L4, each segmental artery was identified bilaterally in more than 90% of subjects, but was identified in less than 10% of patients at L5 [17]; a phenomenon which we also identified.

Zone 3 of L1-3 (postero-superior corner of the vertebral body) is a safe area for extrapedicular puncture. However, this study also showed that an oblique lumbar artery on the side of L4 and L5 vertebrae (Fig. 5F). Previous studies have also confirmed that the L4 and 5 have high variation rates [17]. At this levelwe suggest that CTA of the lumbar artery could be performed to confirm the course of lumbar artery or transpedicular puncture should be used.

This study has the following limitations: (1) CTA can only display the main trunk of the lumbar artery with a diameter of more than 1 mm, and is usually unable to identify more distal branches; (2) Due to the small sample size of this study, confidence is limited about minor anatomical variations; (3) Measurement errors are possible with two observers, however, images were evaluated by a professional imaging physician and an orthopedic specialist, with excellent inter-rater agreement (data not shown). (4) The course of lumbar veins and venous plexus anatomy was not studied in this research. We could not find any report on venous injury during vertebroplasty. The possible reason that is hematoma formed after venous injury is difficult to detect due to the absence of obvious clinical symptoms.

## Conclusion

This study suggests that the posterior superior angle of L1-3 vertebral bodies (zone 3) is usually a safe area with minimal arterialrisk. On this basis, we consider an osseous puncture point within this area, however its clinical safety should be further confirmed in surgical practice. Due to the existence of branches of the lumbar artery at L4 and L5 levels, we advocate caution for this method here.

## Data Availability

The datasets used and/or analyzed during the current study are available from the corresponding author on reasonable request.

## Abbreviations

LAs: Lumbar arteries
CTA: Computed tomographic angiography
PKP: Percutaneous vertebroplasty
OVCF: Osteoporotic vertebral compression fractures
OLIF: Lumbar artery in oblique lumbar interbody fusion
XLIF: Extreme lateral interbody fusion
PVP: Percutaneous Vertebroplasty
TAE: Transarterial Embolization
